# Psychometric evaluation of the Adherence to Refills and Medications Scale (ARMS) in Australians living with gout

**DOI:** 10.1007/s10067-024-07050-y

**Published:** 2024-07-15

**Authors:** Marcel Schulz, Richard O. Day, Matthew J. Coleshill, Nancy E. Briggs, Eindra Aung

**Affiliations:** 1 St Vincent’s Clinical Campus, School of Clinical Medicine, UNSW, Sydney, NSW Australia; 2 Department of Clinical Pharmacology & Toxicology, St Vincent’s Hospital Sydney, Victoria Street, Darlinghurst, NSW 2010 Australia; 3 Black Dog Institute, Sydney, NSW Australia; 4 Stats Central, Mark Wainwright Analytical Centre, UNSW, Sydney, Australia; 5 Northern Sydney Local Health District, NSW Health, Sydney, NSW Australia; 6 Kolling Institute, Pain Management Research Institute, The University of Sydney, Sydney, NSW Australia

**Keywords:** Adherence to Refills and Medications Scale (ARMS), Gout, Medication adherence, Reliability, Urate-lowering therapy (ULT), Validity

## Abstract

This study aimed to examine psychometric properties of the Adherence to Refills and Medications Scale (ARMS) in people with gout. We conducted exploratory factor analysis (EFA) and tested internal consistency (ordinal and Cronbach’s alpha coefficients) and agreement (intraclass correlation coefficient (2,1)) in ARMS scores across three timepoints (baseline, 6, and 12 months) in 487 people with gout. The Kruskal–Wallis test, Spearman’s rank, Kendall’s tau-b correlations, and logistic regression were used to examine the criterion-related validity of the ARMS and factors associated with the ARMS. EFA suggested a one-factor structure, explaining 43.2% of total variance. High internal consistency (ordinal alpha = 0.902 at baseline) and moderate agreement in ARMS scores over time (ICCs > 0.5; *p* < 0.001) were observed. Lower ARMS scores (indicating better adherence) predicted achieving target serum urate (OR, 0.89; 95% CI, 0.83–0.95; *p* < 0.001), but not urate-lowering therapy (ULT) adherence (Proportion of Days Covered (PDC) ≥ 80%) (OR, 0.93; 95% CI, 0.81–1.05; *p* = 0.261). Negative correlations between ARMS and PDC were not statistically significant (Kendall’s tau-b, *r* =  − 0.126, *p* = 0.078; Spearman’s rho =  − 0.173, *p* < 0.073). Differences in median ARMS scores (IQR) of 16 (14–20), 13 (12–15), and 17.5 (15–21) in three groups of participants who reported (1) not taking ULT, (2) taking ULT and adherent, and (3) taking ULT but not adherent, respectively, were statistically significant (*p* < 0.001). Age was the only patient factor independently associated with optimal adherence (ARMS score = 12) (OR, 1.91; 95% CI, 1.50–2.43; *p* < 0.001). The ARMS is a reliable and valid measure of medication adherence behaviours in people with gout, justifying its use in gout medication adherence research.

**Supplementary Information:**

The online version contains supplementary material available at 10.1007/s10067-024-07050-y.

**Table:** 

**Key Points** *• Valid, practical, and efficient methods of measuring adherence to medications are needed in people with gout.* *• Commonly used medication adherence questionnaires have limited validity or have not been validated in people with gout.* *• The Adherence to Refills and Medications Scale (ARMS) has been proven valid and practical in many chronic illnesses but has not been validated in people with gout.* *• We showed the ARMS is valid and reliable for use in people with gout.*		

## Introduction

Gout is the most common form of inflammatory arthritis in men [1]. Gout is caused by elevated serum urate (SU) concentrations leading to urate precipitation in joints, triggering recurrent painful gout flares [2]. Untreated, it can cause joint damage, disability, reduced quality of life, and increased risk of cardiovascular disease and mortality [3, 4]. Successful gout management requires the lifelong use of urate-lowering therapies (ULT). Lowering SU concentrations below the saturation point of urate (< 0.36 mmol/L) promotes dissolution within the affected joints and reduces the risk of urate precipitation [5]. ULT removes the causative agent of flares and thus essentially ‘cures’ a person from the disease [5]. However, despite the availability of safe, generally well-tolerated, and effective ULT for gout [6], adherence to ULT remains sub-optimal [7].

Given the importance of ULT in gout management, tools are required to identify patients with gout at the greatest risk of non-adherence. Subjective measures of adherence, such as questionnaires, can help identify reasons for non-adherence, are relatively simple to use, and are less expensive than more objective measures [8]. However, the Medication Adherence Report Scale (MARS-10) [9] and the Morisky Medication Adherence Scale (MMAS-8) [10] that have been utilised to examine medication adherence in gout have either limited validity or have not been validated specifically for their use in gout.

The Adherence to Refills and Medications Scale (ARMS) is a valid and reliable medication adherence scale designed for use in chronic disease populations and patients with low literacy [11]. It was highly correlated with a commonly used self-reported adherence measure (Medication Adherence Questionnaire) [12] and a measure of refill adherence (cumulative medication gap) [11]. To date, the ARMS has been validated for use across numerous languages and chronic diseases, including diabetes, coronary heart disease, and breast cancer, but not in gout [13–17]. ARMS notably assessed patients’ ability to take and refill their prescribed medications [11]. ARMS was designed for use in patients with low literacy, and simple wording was used [11]. Previous studies reported that the ARMS exhibited a high internal consistency (Cronbach’s alpha ranged from 0.74 to 0.954) [11, 13, 15–20]. Regarding criterion-related validity, the ARMS was associated with a self-reported adherence measure developed by Morisky and colleagues [11], medication refill adherence during the previous 6 months [11], glycaemic control [13, 18], and blood pressure control [15, 16].

We explored the psychometric properties of the ARMS in people living with gout. Specifically, we aimed to examine whether the ARMS’s two-factor structure (i.e. the medication-taking behaviour and refill behaviour subscales) applies to people with gout. We also examined the internal consistency and agreement in ARMS scores across three timepoints (baseline, 6, and 12 months). The value of the ARMS as a predictor of adherence in general and ULT adherence specifically, as well as achievement of target serum urate (SU), was also examined.

## Materials and methods

### Study design and participants

This study utilised data from the Gout APP (GAPP) trial [21], a 12-month randomised-controlled trial (RCT) examining the effectiveness of a mobile app designed to help people self-manage their gout and achieve the target SU concentration (≤ 0.36 mmol/L) by adhering to ULT. Participants were randomised to receive either an intervention or control app to use for 12 months. Participants completed surveys and blood tests for SU at baseline, 6, and 12 months.

### Inclusion and exclusion criteria

Eligible participants were over 18 years of age, residents of Australia, diagnosed with gout, reported having at least one gout flare in the preceding 12 months, were receiving or eligible to start/restart ULT, and had access to a smartphone or tablet device with internet access. Exclusions included insufficient technological skills to use the mobile app, limited understanding of English, or a psychological condition precluding participation.

### Ethical considerations

The GAPP trial was approved by the University of New South Wales’s Human Research Ethics Committee (HC15199 and HC210543). All participants provided written informed consent.

### The adherence to refills and medications scale

The ARMS is a 12-item ordinal scale developed to measure general medication adherence behaviour [11]. There are two subscales: eight items comprising the ‘medication-taking’ subscale which measures a person’s ability to take prescribed medications as directed and four items comprising the prescription-refill subscale that evaluates a person’s adherence acquiring prescription refills. Each item is rated on a four-point Likert scale: 1 (none of the time), 2 (some of the time), 3 (most of the time), and 4 (all of the time). Responses are summed to produce an overall adherence score ranging from 12 to 48, with a higher score indicating poorer general medication adherence.

### Self-reported ULT-taking status and daily doses of ULT

The GAPP survey asked participants to report whether they were taking any medication for their gout at the time of the survey and the name, strength, daily dose, and frequency (e.g. once a day) of gout medications. The survey also asked a multi-response question: ‘If there are times that you do not take your medications for your gout, what is/are the reason(s) for this? Please select all that apply. If a participant who reported taking ULT, selected ‘I always take my medications’ as the only one of the 5 options to the multi-response question, this was considered ‘self-reported adherence to ULT’. The self-reported ULT-taking status was categorised as follows: (1) ‘not taking ULT’, (2) ‘taking ULT and adherent’, and (3) ‘taking ULT but not adherent’.

### Estimating ULT adherence

Adherence to ULT was estimated [22] from the Pharmaceutical Benefits Scheme (PBS) dispensing claims data using Proportion of Days Covered (PDC) and patient-reported daily doses [23]. Participants in the GAPP trial optionally consented for the release of their PBS claims data covering a 3-year period. PBS data was supplied by Services Australia and included information on all dispensings for each participant in this 3-year period with the dates of dispensings, formulation strength, and number of tablets supplied. Briefly, the baseline PDC was estimated in the approximately 12-month period before the link to the app was sent to participants. The PDC was calculated in participants who reported taking, and were dispensed, any ULT drug (allopurinol or febuxostat) using the formula:

$$PDC=\frac{\text{Total days covered given the estimated daily dose}}{\text{Total number of days from first to last ULT dispensing}}\times 100.$$

Participants with PDC values ≥ 80% were considered adherent [24]. More information on the methodology used to estimate the PDC can be found in Online Resource 1, available at *Rheumatology* online.

### Statistical analyses

Data were analysed using SPSS statistical software (IBM SPSS Statistics 27.0, Chicago, IL), with a level of significance set at 0.05 and all confidence intervals (CIs) set at 95%. Ordinal alpha coefficients were computed using SAS software (version 9.4, the SAS System for Windows, SAS Institute Inc., Cary, NC, USA). Descriptive analyses of patient characteristics and ARMS item scores and exploratory analyses to evaluate missing data and examine score distribution and item response patterns (floor or ceiling effects) were conducted.

We compared baseline characteristics between participants who completed the ARMS at all three timepoints and those who did not. Baseline differences were evaluated using appropriate statistical tests, including Pearson’s chi-square test, independent samples *t*-test, or its non-parametric equivalent where applicable.

### Construct validity

As the ARMS scale was ordinal and its distribution skewed, polychoric correlation matrices were computed to evaluate the scale’s construct validity [25]. A free and comprehensive POLYMAT-C program for SPSS was used for the computation of polychoric correlations [26]. Polychoric correlation coefficients were chosen in preference to Pearson’s correlation coefficients as the polychoric correlation method has been demonstrated to produce unbiased parameter estimates for exploratory factor analysis (EFA) and more accurate estimations of dimensionality than other methods using ordinal variables [25, 27].

To ensure the suitability of the item pool for factor analysis, Kaiser–Meyer–Olkin’s test and Bartlett’s test of sphericity were computed [28]. The appropriateness of inter-item correlation matrices was explored by visual examination to ensure that coefficients were above 0.30 but below 0.90 [29]. To determine the number of factors to be retained in a subsequent EFA, as recommended [28], a combination of parallel analysis (PA) and Velicer’s minimum average partial (MAP) test was utilised, with scree plots reserved as a useful addition to make the final decision. EFA was performed to test the scale’s structure.

We used multiple imputation (MI) to address missing ARMS data. We imputed 25 datasets assuming either missing at random (MAR) or missing not at random (MNAR) scenarios using the mice (Multivariate Imputation by Chained Equations) package in R (version 4.4.0). The predictive mean matching method was used for MAR [30]. The pattern-mixture model method was used for MNAR [31, 32] to allow for the assumption that participants with missing data at follow-up who had sub-optimal adherence at baseline (ARMS score > 12) also had sub-optimal adherence at follow-up. Imputed item responses for these participants were adjusted by adding + 0.25 at each follow-up imputed item response value [31, 32]. If the imputed item response score was 4 (the highest item score possible), it was left as 4 in the dataset. Next, a polychoric correlation matrix was computed for each imputed dataset and an EFA was performed forcing a one-factor structure. Finally, the package mifa (Multiple Imputation for Factor Analysis) was used to calculate the proportion of variance accounted for by the one factor [30], and the factor loadings were extracted to examine the minimum, median, and maximum values for the 25 datasets.

### Internal consistency, agreement, and responsiveness

Internal consistency reliability of the ARMS was assessed using ordinal alpha and Cronbach’s alpha coefficients at baseline, 6, and 12 months. A coefficient ≥ 0.70 was considered acceptable [33]. The developers of the ARMS found it to have an acceptable internal consistency according to Cronbach’s alpha coefficient [11], the use of which in ordinal scales, however, was disputed [34]. As such, the ordinal alpha was calculated [35]. To assess the agreement of the scale over time, we examined intraclass correlation coefficients (ICC, 2,1) between the baseline and each of the two follow-up timepoints: 6 and 12 months. The ICC form of ‘2,1’ was chosen as it measures absolute agreement using two-way random effects and assumes a single measurement at each timepoint for each participant [36]. The ability of ARMS to detect change in medication adherence over time (responsiveness) was assessed in all participants from baseline to 12 months using a Wilcoxon Signed-Ranks test.

### Criterion-related validity and predictors of optimal medication adherence

After finding an optimal scale structure, the criterion-related validity of the scale was examined using data from all participants who completed ARMS at baseline. ARMS scores were dichotomised at the lowest score of 12, considering optimal medication adherence behaviour. We separately examined the association of baseline ARMS scores and optimal adherence (ARMS score = 12), respectively, with baseline target SU (≤ 0.36 mmol/L) using multivariable logistic regression adjusting for age and sex. These analyses were repeated with claims-data-derived ULT adherence (PDC ≥ 80%) at baseline as the outcome variable.

A Kruskal–Wallis test was performed to examine whether there was a difference in ARMS scores between the three categories of self-reported ULT-taking status.

Lastly, a forward stepwise binary logistic regression procedure was used to identify predictors of optimal adherence (ARMS score = 12). A criterion of *p* < 0.1 was required for variables to be evaluated in the multivariable model, but to be retained in the final model, the criterion required was *p* < 0.05.

Additionally, we assessed the criterion-related validity of the ARMS and examined factors associated with optimal adherence at baseline for those who completed the ARMS at all three timepoints.

## Results

### Baseline demographic and clinical characteristics

Among 537 trial participants, 487 completed the ARMS questionnaire at baseline, 372 at 6 months, and 352 at 12 months. The mean age (SD) of those 487 participants was 57.5 (12.8), range 22–90 years, and 95.5% were male (Table 1). The cohort was predominantly Caucasian (as the sole ancestry) (82.9%), currently employed or a student (66.8%), and held a university bachelor’s degree or higher (74.9%). The median body mass index (BMI) was 30 (interquartile range (IQR), 27–33), about half were in the obese category (BMI > 30), and 41.3% were categorised as overweight. The median number (IQR) of comorbid conditions was 1 (0–3).

**Table 1:** Sociodemographic and clinical characteristics of participants at baseline (*n* = 487)

Patient factors	Overall	Optimal adherence (ARMS score = 12)	Sub-optimal adherence (ARMS score > 12)	*p* value
Age in years: mean (SD)	57.5 (13)	65 (11)	56 (13)	< 0.001
Age				
Below 65	334 (68.6)	43 (45.3)	291 (74.2)	< 0.001
65 and above	153 (31.4)	52 (54.7)	101 (25.8)	
Sex				
Male	465 (95.5)	87 (91.6)	378 (96.4)	0.053
Female	22 (4.5)	8 (8.4)	14 (3.6)	
Language spoken at home				
English	454 (94.6)	90 (94.7)	364 (94.5)	0.941
Language other than English	26 (5.4)	5 (5.3)	21 (5.5)	
Ancestry				
White/Caucasian/European (reported as the sole ancestry)	398 (82.9)	87 (91.6)	311 (80.8)	0.012
Others	82 (17.1)	8 (8.4)	74 (19.2)	
Work status				
Employed or student	320 (66.8)	50 (52.6)	270 (70.3)	< 0.001
Unemployed	23 (4.8)	2 (2.1)	21 (5.5)	
Retired	136 (28.4)	43 (45.3)	93 (24.2)	
BMI: median (IQR)	30 (27–33)	30 (26–32)	29 (27–34)	0.035
BMI (categorised)				
Normal and underweight	54 (11.3)	15 (15.8)	39 (10.1)	0.108
Overweight	198 (41.3)	43 (45.3)	155 (40.3)	
Obese	228 (47.5)	37 (38.9)	191 (49.6)	
Current living arrangements				
Couples and non-couples with no co-dependents	293 (62.7)	68 (72.3)	224 (60.3)	0.03
Couples and non-couples with co-dependents	174 (37.3)	26 (27.7)	148 (30.7)	
Annual income				
< $41,600	58 (15.7)	12 (18.2)	46 (15.1)	0.486
$41,600–$103,999	159 (43)	31 (47)	128 (42.1)	
≥ $104,000	153 (41.4)	23 (34.8)	130 (42.8)	
Number of comorbidities: median (IQR)	1 (0–3)	2 (1–3)	1 (0–2)	< 0.001
Binge-drinking behaviour: How many standard drinks consumed on the largest occasion? Median (IQR)	6 (3–12)	6 (3–10)	8 (4–12)	0.037
Have seen a GP in the past 6 months?				
Not seen a GP	22 (4.5)	5 (5.3)	17 (4.4)	0.783
Seen a GP	463 (95.5)	90 (94.7)	373 (95.6)	
Have seen a GP for gout in the past 6 months?				
Not seen a GP for gout	84 (17.3)	25 (26.3)	59 (15.1)	0.01
Seen a GP for gout	401 (82.7)	70 (73.7)	331 (84.9)	
Have seen the specialist for gout in the past 6 months?				
Not seen a rheumatologist for gout	405 (83.5)	75 (78.9)	330 (84.6)	0.182
Seen a rheumatologist for gout	80 (16.5)	20 (21.1)	60 (15.4)	
Number of gout attacks in the past 6 months: Median (IQR)	3 (1.75–4)	2 (1–4)	3 (2–4)	0.043
Reported taking ULT?				
No	182 (37.4)	30 (31.6)	151 (38.5)	0.209
Yes	305 (62.6)	65 (68.4)	241 (61.5)	
Number of tophi: Median (IQR)	0 (0–1)	0 (0–1)	0 (0–1)	0.826
Most recent gout attack pain (scales 1–10): median (IQR)	7 (5–8)	7 (5–8)	7 (5–8)	0.209
Number of days since last gout attack: median (IQR)	27 (8–84)	45 (7–115)	25 (8–75)	0.116
Serum urate concentration (mmol/L): mean (SD)	0.43 (0.14)	0.40 (0.10)	0.44 (0.10)	0.002
Target serum urate (SU ≤ 0.36 mmol/L)				
Not achieved	366 (75.5)	59 (62.1)	307 (78.7)	< 0.001
Achieved	119 (24.5)	36 (37.1)	83 (21.3)	
Smoking status				
Not smoking	442 (91.9)	90 (94.7)	352 (91.2)	0.257
Smoking	9 (8.1)	5 (5.3)	34 (8.8)	
Education				
Year 12 and below	121 (25.1)	22 (23.2)	99 (25.6)	0.625
Tertiary education	361 (74.9)	73 (76.8)	288 (74.4)	
Baseline PDC (%): mean (SD)	83 (21)	87 (16)	82 (21)	0.366
Baseline ULT adherence				
Adherent (PDC ≥ 80%)	69 (63.9)	10 (66.7)	59 (63.4)	
Non-adherent (PDC < 80%)	39 (36.1)	5 (33.3)	34 (36.6)	0.809

*BMI* body mass index, *GP* general practitioner, *IQR* interquartile range, *SD* standard deviation, *SU* serum urate, *ULT* urate-lowering therapy, *PDC* Proportion of Days Covered

All results are presented in *n* (%) unless otherwise indicated. Patients who responded either ‘I do not know’ or ‘I would rather not respond’ were treated as missing values

The mean SU concentration (SD) of the cohort was 0.43 (0.14) mmol/L, with 25% at or below the recommended target SU (≤ 0.36 mmol/L) at baseline. Almost two-thirds (63%) reported taking a ULT at the time of the baseline survey, and 182 (37%) reported not taking ULT. Of those who reported taking ULT, 144 (30%) reported they were adherent and 161 (33%) reported they were not adherent.

### ARMS data

Among 487 participants who completed the ARMS at baseline, 368 (75.6%) completed the scale again at 6 months, 345 (70.8%) at 12 months, and 311 (63.9%) at all timepoints.

**Fig. 1:**
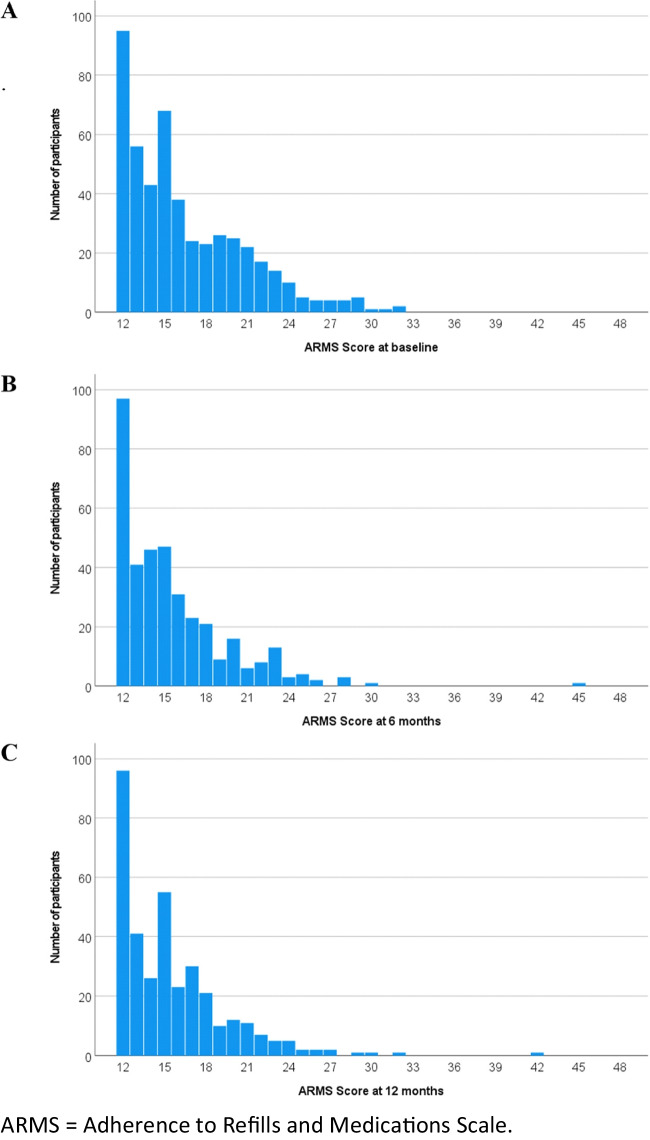
Distribution of ARMS scores at **A** baseline (*n* = 487), **B** 6 months (*n* = 372), and **C** 12 months (*n* = 352). ARMS, Adherence to Refills and Medications Scale

The ARMS scores were positively skewed at all timepoints (Fig. 1). The mean (SD) and median (IQR) ARMS scores were 16 (4) and 15 (13–19) at baseline, 16 (4) and 15 (12–17) at 6 months, and 16 (4) and 15 (12–17) at 12 months, respectively.

A ‘floor effect’ was observed in all 12 items, which were scored ‘1’ by 45 to 93% of participants. Item 12 “How often do you plan ahead and refill your medicines before they run out?” was scored ‘1’ (reverse-coded ‘all of the time’) by 45% at baseline, 52% at 6 months, and 51% at 12 months. Item 11 “How often do you put off refilling your medicines because they cost too much money?” was scored ‘1’ (none of the time) by 93% at all timepoints. However, only 19.5%, 26.1%, and 27.3% of participants at baseline, 6, and 12 months, respectively, had an ARMS score of 12 (the lowest possible score—optimal adherence). No ceiling effect was observed as the items were scored ‘4’ by only 0.2% to 11% of participants. Regarding attrition over time (Supplementary Table S1), the median (IQR) ARMS score in participants who completed the ARMS at all timepoints (*n* = 311) was slightly lower than in participants who did not (*n* = 176) (15 (13–19) versus 16 (13–20); *p* = 0.021). In addition, participants who completed the ARMS at all timepoints were more likely to be retired (*p* = 0.034), have slightly lower BMI (*p* = 0.039), and report longer duration since their last gout flare (*p* = 0.001).

### Construct validity

Inspection of the inter-item polychoric correlation matrix at each timepoint revealed that most items had some correlation with each other, and multicollinearity was not evident as correlations did not exceed 0.8. The Kaiser–Meyer–Olkin measure of sampling adequacy was 0.89 and Bartlett’s test of sphericity (BT 2875.041) was statistically significant (*p* < 0.0001). These results indicated that the data were suitable for conducting an EFA.

Results from parallel analysis (PA) and minimum average partial (MAP) tests in addition to factor scree plots at each timepoint demonstrated that a one-factor structure would be optimal. Thus, EFA was performed using principal axis factoring (PAF) with no rotation and forcing a one-factor structure. As all items had a factor loading above 0.5 (Table 2), it was decided to retain all items. One factor accounted for 43.2% of the variance and had an eigenvalue of 5.188. A two- or three-factor solution did not result in a clear separation of the items.

**Table 2:** Factor loadings from principal axis factoring with no rotation and forcing a one-factor structure at each timepoint

ARMS items	Factor loadings		
	Baseline (*n* = 487)	6 months (*n* = 372)	12 months (*n* = 352)
	Factor 1	Factor 1	Factor 1
1: How often do you forget to take your medicine?	0.638	0.563	0.524
2: How often do you decide not to take your medicine?	0.595	0.586	0.640
3: How often do you forget to get prescriptions filled?	0.737	0.776	0.669
4: How often do you run out of medicine?	0.712	0.811	0.760
5: How often do you skip a dose of your medicine before you go to the doctor?	0.787	0.829	0.886
6: How often do you miss taking your medicine when you feel better?	0.732	0.661	0.730
7: How often do you miss taking your medicine when you feel sick?	0.660	0.748	0.710
8: How often do you miss taking your medicine when you are careless?	0.722	0.654	0.691
9: How often do you change the dose of your medicines to suit your needs?	0.505	0.522	0.640
10: How often do you forget to take your medicine when you are supposed to take it more than once a day?	0.700	0.704	0.728
11: How often do you put off refilling your medicines because they cost too much money?	0.507	0.652	0.683
12: How often do you plan ahead and refill your medicines before they run out?	0.511	0.446	0.394

ARMS Adherence to Refills and Medications Scale

EFA performed on 25 imputed datasets showed that median factor loadings exceeded 0.3 at all timepoints, and the aggregated mean estimates for variance in item responses explained by the one factor were above 30% under either missingness mechanism (MAR or MNAR) (Supplementary Tables S2 and S3). No evidence suggested that a one-factor solution was not optimal when considering the missing data mechanism as either MAR or MNAR.

### Internal consistency, agreement, and responsiveness

Ordinal alpha coefficients were 0.902 at baseline, 0.903 at 6 months, and 0.907 at 12 months, indicating high internal consistency. In comparison, Cronbach’s alpha coefficients were 0.790 at baseline, 0.773 at 6 months, and 0.773 at 12 months, indicating acceptable internal consistency. The intraclass correlation coefficient (ICC, 2,1) indicated an overall moderate agreement in ARMS scores across timepoints (ICC, baseline to 6 months = 0.518, *p* < 0.001; ICC, baseline to 12 months = 0.573, *p* < 0.001). A Wilcoxon Signed-Ranks test indicated that the ARMS score (mean rank = 123.56) at 12 months was significantly lower than the ARMS score at baseline (mean rank = 136.72), with a *Z*-score of − 3.584 and a *p*-value of < 0.001.

### Criterion-related validity

Criterion-related validity of the ARMS was first examined in relation to achieving the target SU and ULT adherence (PDC). For every point increase in the ARMS score (indicating poorer adherence), the odds of achieving the target SU decreased by 11% (odds ratio (OR) adjusted for age and sex, 0.89; 95% CI, 0.84–0.95; *p* < 0.001). Participants with optimal adherence were almost twice as likely to achieve target SU than those with sub-optimal adherence (OR, 1.94; 95% CI, 1.17–3.21; *p* < 0.05; Table 3).

**Table 3:** Association between the ARMS score and target serum urate at baseline

Adherence level (ARMS score)	At target SU concentration (SU ≤ 0.36 mmol/L)		Adjusted OR (95% CI)^a^
	Yes, *n* (%)	No, *n* (%)	
Sub-optimal (> 12)	83 (69.7)	307 (83.9)	1 (ref)
Optimal (= 12)	36 (30.3)	59 (16.1)	1.94 (1.17–3.21)*
**ARMS score**	–	–	0.89 (0.84–0.95)**

*ARMS* Adherence to Refills and Medications Scale, *SU* serum urate

^a^Adjusted for age and sex

^*^p < 0.05, **p < 0.01

In 108 participants whose baseline PDC data was available, no significant association was found between the ARMS score and ULT adherence (PDC ≥ 80%) (adjusted OR, 0.93; 95% CI, 0.81–1.05; *p* = 0.261) or between optimal adherence (ARMS score = 12) and ULT adherence (adjusted OR, 1.05; 95% CI, 0.31–3.54; *p* = 0.932). Similarly, a weak negative correlation between baseline ARMS scores and baseline PDCs was not statistically significant (Kendall’s tau-b, *r* =  − 0.126, *p* = 0.078; Spearman’s rho =  − 0.173, *p* = 0.073).

A Kruskal–Wallis test showed that there was a significant difference (*p* < 0.001) in ARMS scores between three groups of self-reported ULT-taking status: (1) ‘not taking ULT’, (2) ‘taking ULT and adherent’, and (3) ‘taking ULT but not adherent’, with median ARMS scores (IQR) of 16 (14–20), 13 (12–15), and 17.5 (15–21) in these groups, respectively (Table 4).

**Table 4:** ARMS scores by ‘self-reported ULT-taking status’ at baseline

Self-reported ULT-taking status	Median ARMS score (IQR)	*p* value across groups
Not taking ULT	16 (14–20)	< 0.001^a^
Taking ULT and adherent	13 (12–15)	
Taking ULT but not adherent	17.5 (15–21)	

*ARMS* Adherence to Refills and Medications Scale, *ULT* urate-lowering therapy

^a^Using Kruskal Wallis Test

Similar results were found when repeating these analyses in the 311 participants who completed the ARMS at all timepoints. Participants with optimal adherence were more than twice as likely to achieve target SU than those with sub-optimal adherence (OR adjusted for age and sex, 2.43; 95% CI, 1.31–4.53; *p* < 0.05). Using a Kruskal–Wallis test, the lowest median ARMS score (IQR) (13 (12–14)) was also observed in the ‘taking ULT and adherent’ group, while the highest (17 (15–21)) was observed in the ‘taking ULT but not adherent’ group (*p* < 0.001).

### ULT adherence

In 108 participants who had two or more ULT dispensings in the 12-month period before the app link was sent to them, a PDC had been calculated using self-reported dosing data from the baseline survey along with the PBS claims data of dispensings [22]. These 108 people comprised 101 taking allopurinol and 7 taking febuxostat. The mean (SD) and median (IQR) PDCs were 83% (21%) and 92% (70–100%), respectively, with 70 (64.8%) categorised as being adherent to ULT (PDC ≥ 80%).

### Predictors of optimal medication adherence behaviour

According to the univariable logistic regression analysis, participants had greater odds of having optimal medication adherence behaviour (ARMS score = 12) at baseline if they were older (OR, 1.96; 95% CI, 1.57–2.43; *p* < 0.001), Caucasian (OR, 2.59; 95% CI, 1.20–5.57; *p* = 0.015), retired (OR, 2.50; 95% CI, 1.56–4.00; *p* < 0.001), reported a greater number of comorbidities (OR, 1.26; 95% CI, 1.11–1.42; *p* < 0.001), and lower odds if they had no co-dependents (OR, 0.58; 95% CI, 0.35–0.96; *p* = 0.032), and had not seen a GP in last 6 months (OR, 0.50; 95% CI, 0.29–0.85; *p* = 0.011). However, age was the only variable which remained in the multivariable model, showing that the odds of having the optimal adherence (ARMS score = 12) increased by 91% (OR, 1.91; 95% CI, 1.50–2.43; *p* < 0.001) for every 10-year increase in age (Table 5).

**Table 5:** Patient factors associated with optimal adherence (ARMS score = 12) at baseline

Patient factors	Univariable analysis		Multivariable analysis^a^	
	OR (95% CI)	*p* value	OR (95% CI)	*p* value
White/Caucasian/European as the sole ancestry	2.59 (1.20–5.57)	0.015		
Age (for every 10 years)	1.96 (1.57–2.43)	< 0.001	1.91 (1.50–2.43)	< 0.001
Employment status				
Employed or student	1 (ref)	< 0.001		
Unemployed	0.51 (0.12–2.26)			
Retired	2.50 (1.56–4.00)			
Number of comorbidities	1.26 (1.11–1.42)	< 0.001		
Binge-drinking behaviour	0.95 (0.91–0.99)	0.016		
Living with co-dependents	0.58 (0.35–0.96)	0.032		
Seen GP for gout in last 6 months	0.50 (0.29–0.85)	0.011		

*ARMS* Adherence to Refills and Medications Scale, *CI* confidence interval, *GP* general practitioner, *OR* odds ratio

^a^Variables included in the multivariable model were ‘BMI (body mass index)’, ‘White/Caucasian/European as the sole ancestry’, ‘Age (for every ten years)’, ‘Employment status’, ‘Number of comorbidities’, ‘Binge-drinking behaviour’, ‘Living with co-dependents’, and ‘Seen GP for gout in last 6 months’. Only ‘Age’ remained in the final model

Sensitivity analyses among participants who completed the ARMS at all timepoints showed that participants had greater odds of having optimal medication adherence behaviour (ARMS score = 12) at baseline if they were older (OR, 1.71; 95% CI, 1.28–2.29; *p* < 0.001) or reported a greater number of comorbidities (OR, 1.23; 95% CI, 1.11–1.47; *p* = 0.001), and lower odds if they had a higher BMI (OR, 0.47; 95% CI, 0.25–0.89; *p* < 0.05) or were unemployed (OR, 0.40; 95% CI, 0.22–0.70; *p* < 0.05). Age remained significant in the multivariable model (OR, 1.71; 95% CI, 1.28–2.29; *p* < 0.001), as well as the number of comorbidities (OR, 1.25; 95% CI, 1.06–1.47; *p* = 0.007) and BMI (OR, 0.93; 95% CI, 0.87–0.99; *p* = 0.032).

## Discussion

ULT is key to reducing urate concentrations in people with gout, thereby avoiding further gout flares; however, adherence to ULT is notably poor. Recent systematic reviews indicate that adherence to ULT assessed from prescription refills recorded in claims databases is as low as 10% and as high as 47% [37, 38]. This is generally lower than other chronic diseases including hypertension (53–71%) [39], diabetes mellitus (20–88%) [40], and rheumatoid arthritis (9.3–94%) [41]. In primary care, studies have found that between 25 and 73% of patients with gout were prescribed ULT, while only between 41 and 70% achieved the target SU concentration [42–46]. Factors contributing to poor ULT adherence have been identified. Gout is stigmatised as a disease of opulence and widely believed to be controllable with diet and lifestyle modifications which negatively impacts adherence to medications [47]. People with gout may stop ULT due to the long periods without flares [48]. Further, the risk of acute gout flares with ULT initiation induces poor adherence, which emphasises the importance of effective education of people commencing ULT and provision of flare prophylaxis upon ULT initiation [7, 49]. For successful treatment with ULT, patients and health professionals need to understand the importance of monitoring SU concentrations to titrate to an appropriate ULT dose and maintain SU concentrations below the target level [46, 50, 51]. As demonstrated from our study on the association between the ARMS and achievement of target SU, the ARMS may be a valuable tool in identifying patients at greatest risk of not reaching target SU.

The ARMS has been validated in people with at least one chronic disease [11, 17, 19, 20, 52], hypertension [15, 16], and type 2 diabetes mellitus [13, 18]. Consistent with this, our findings confirm that the ARMS is a valid and reliable measure of the ability to correctly self-administer and refill prescriptions of prescribed medications in people with gout. Interestingly, the ARMS appeared to measure these two medication adherence behaviours as a single construct.

In the validation study of the original version of ARMS [11], the two-factor analysis resulted in factor 1 accounting for 35.1% and factor 2, 10% of variance. When combined, almost as much variance was explained (45.1%) as our study’s one factor (43.2%). A two-factor solution was also reported in six studies validating the ARMS in other languages and an adapted version for measuring adherence to diabetes medicines [15–20, 52]. On the other hand, the validation of the Korean version in adults with type 2 diabetes [13] demonstrated that 3 dimensions explained 54.7% of the total variance. Our results indicate a one-factor structure was optimal, and a two- or three-factor solution did not result in clear item separation. Our findings may be explained by the characteristics of our cohort, our choice of conducting factor analysis using polychoric inter-item correlations, using a parallel analysis (PA) criterion [53], and undertaking a minimum average partial (MAP) test [28]. Summated rating scales with Likert-type response items, such as the ARMS, produce scores that are ordinal in nature and often highly skewed. When such scales are used, it has been recommended that factor analyses should be conducted on the matrix of polychoric inter-item correlations instead of Pearson correlations for more accurate models [25]. Interestingly, only one [20] of the nine previous validation studies [11, 13, 15–19, 52] used either the PA or MAP methods to decide how many factors to extract in the factor analysis.

All ordinal alpha coefficients were > 0.9, indicating high internal consistency [33]. This finding is consistent with the validation study of the original version (Cronbach’s alpha = 0.814) [11] and validation studies in other populations [13, 15–20, 52]. Since a Pearson covariance matrix is routinely used in the calculation of Cronbach’s alpha which assumes that the data is continuous [54], it may be best to report alternatives to Cronbach’s alpha in examining the internal consistency of the ARMS. Unlike the previous studies mentioned, we observed a moderate agreement in ARMS scores over time [36], and a decrease in ARMS scores between baseline and 12 months. This was expected as the intervention, or even participation, in the GAPP trial might have altered the ARMS scores in participants over time.

Previous research has associated the ARMS with the MMAS-8, blood pressure control [15, 16], and glycaemic control [11, 13, 18, 19]. In our study, participants were almost twice as likely to have achieved target SU if they had optimal adherence (ARMS score = 12) than those with sub-optimal adherence. Our study also demonstrated the association of ARMS with self-reported ULT-taking status as the median ARMS score was near-optimal in participants who reported taking ULT and being adherent to ULT. These participants were more likely to have a better medication adherence behaviour (lower ARMS scores) compared to participants who did not report taking ULT or participants who reported taking ULT but not being adherent to ULT.

One source of weakness in this study which we believe overall decreased the ARMS scores in participants over time was the number of participants lost to follow-up. Better medication adherence behaviours (lower ARMS scores) among participants who completed the ARMS at all timepoints (compared to those who did not) may partly be attributed to differences in patient characteristics between these groups. We found that retirees were more likely to complete the survey at all timepoints while older participants reported better medication adherence. However, the factor structure and loadings remained consistent across three timepoints despite attrition at 6-month and 12-month timepoints, and the results from MI analyses indicated that our EFA results were not significantly influenced by either MAR or MNAR mechanisms. In fact, using imputed datasets under MAR and MNAR assumptions yielded factor loadings and item response variances consistent with those obtained from the factor analysis performed on the complete sample.

An advantage of this study is the availability of ARMS data at three timepoints to examine the factor structure and internal consistency of the scale. The results consistently identified the one-factor structure, and calculation of ordinal alpha using polychoric correlations showed high internal consistency at each timepoint. Another strength is the use of an objective clinical measure (SU) for validation, as well as additional adherence measures. Limitations of the study included missing data at follow-up timepoints and the GAPP trial’s selection criteria excluding those without access to a mobile phone (or tablet device) or the Internet, and those with limited English language proficiency.

## Conclusion

The ARMS is a well-validated tool for use in populations with low literacy and in numerous chronic diseases. Our psychometric findings indicate that the ARMS is a valid and reliable tool for measuring the ability to self-administer and refill prescribed medications in people with gout. The 12-item ARMS was able to discriminate between groups with different levels of serum urate concentrations, as well as groups with different self-reported ULT-taking status. Thus, given the low rates of ULT adherence and the central role of ULT in managing gout, the ARMS may be a useful tool for identifying opportunities to improve gout management in people with gout.

### Supplementary Information

Below is the link to the electronic supplementary material.

Supplementary file1 (DOCX 18.1 KB)

Supplementary file2 (DOCX 27.5 KB)

Supplementary file3 (DOCX 26.9 KB)

## Data Availability

Research data are not shared.
