# Supplementary material for: Psychometric evaluation of the Adherence to Refills and Medications Scale (ARMS) in Australians living with gout
Source: Clin Rheumatol. 2024 Jul 15;43(9):2943–54. doi: 10.1007/s10067-024-07050-y (PMC11330394; doi:10.1007/s10067-024-07050-y)
Supplement: Supplementary file 1 — Supplementary file1 (DOCX 18.1 KB) [file 10067_2024_7050_MOESM1_ESM.docx]

**SUPPLEMENTARY INFORMATION 1:**

**Online Resource 1. Proportion of Days Covered (PDC) estimations:**

We utilised a ‘lookback window’ to account for medications on hand from the last dispensing before the period of interest [1]. Daily doses of ULT for each participant were based on the most likely ULT dose prescribed per day over the PDC observation periods using a combination of each participant’s self-reported daily dose and their PBS dispensing records.

Consensus decisions were made and documented by the investigators (MS, EA, MC, RD) for each participant and, when required, additional clinical input from the rheumatologist investigator (RD). Dose estimate confidence for each participant was graded none, low, moderate, or high, depending on the self-reported daily dose’s completeness, plausibility, and alignment with PBS dispensing data. Whenever participants reported a tablet strength not available in Australia (e.g., 450 mg of allopurinol), we checked whether this was plausible given the dispensed tablet size (e.g., 450 mg/day by taking 1.5 x 300 mg tablets/day).

**References:**

1. Loucks, J., et al., *Proportion of days covered as a measure of medication adherence.* American Journal of Health-System Pharmacy, 2021. **79**(6): p. 492-496.
