# Supplementary material for: Psychometric evaluation of the Adherence to Refills and Medications Scale (ARMS) in Australians living with gout
Source: Clin Rheumatol. 2024 Jul 15;43(9):2943–54. doi: 10.1007/s10067-024-07050-y (PMC11330394; doi:10.1007/s10067-024-07050-y)
Supplement: Supplementary file 2 — Supplementary file2 (DOCX 27.5 KB) [file 10067_2024_7050_MOESM2_ESM.docx]

**SUPPLEMENTARY INFORMATION 2:**

**Supplementary Table S1**: Baseline sociodemographic and clinical characteristics of participants who completed the ARMS at all timepoint (n=311) and participants who did not (n=176).

| **Patient factors** | **Completed ARMS at all timepoints**  **(n=311)** | **Not completed ARMS at all timepoints**  **(n=176)** | **P value** |
| --- | --- | --- | --- |
| Age in years: Mean (SD) | 58.1 (13) | 56.3 (14) | 0.107 |
| Age |  |  | 0.163 |
| Below 65 | 205 (65.9) | 159 (71.6) |  |
| 65 and above | 106 (34.1) | 63 (28.4) |  |
| Sex |  |  | 0.058 |
| Male | 300 (96.5) | 207 (92.8) |  |
| Female | 11 (3.5) | 16 (7.2) |  |
| Language spoken at home |  |  | 0.750 |
| English | 294 (94.8) | 161 (94.2) |  |
| Language other than English | 16 (5.2) | 10 (5.8) |  |
| Ancestry |  |  | 0.219 |
| White/Caucasian/European (reported as the sole ancestry) | 48 (15.5) | 137 (80.1) |  |
| Others | 262 (84.5) | 34 (19.9) |  |
| Work status |  |  | 0.034 |
| Employed or student | 194 (62.8) | 128 (74.4) |  |
| Unemployed | 17 (5.5) | 6 (3.5) |  |
| Retired | 98 (31.7) | 38 (22.1) |  |
| BMI: Median (IQR) | 29.3 (27-33) | 30.7 (27-34) | 0.039 |
| BMI (categorised) |  |  | 0.027 |
| Normal and underweight | 39 (12.6) | 15 (8.8) |  |
| Overweight | 138 (44.5) | 61 (35.7) |  |
| Obese | 133 (42.9) | 95 (55.6) |  |
| Current living arrangements |  |  | 0.133 |
| Couples and non-couples with no co-dependents | 196 (65.3) | 98 (58.3) |  |
| Couples and non-couples with co-dependents | 104 (34.7) | 70 (41.7) |  |
| Annual income |  |  | 0.740 |
| <$41,600 | 35 (14.6) | 23 (17.4) |  |
| $41,600-$103,999 | 105 (43.9) | 54 (40.9) |  |
| ≥$104,000 | 99 (41.4) | 55 (41.7) |  |
| Number of comorbidities: Median (IQR) | 1 (0-3) | 1 (0-2) | 0.089 |
| Binge-drinking behaviour: How many standard drinks consumed on the largest occasion? Median (IQR) | 6 (3-12) | 7 (4-12.5) | 0.112 |
| Have seen a GP in the past 6 months? |  |  | 0.624 |
| Not seen a GP | 13 (4.2) | 9 (5.1) |  |
| Seen a GP | 298 (95.8) | 166 (94.9) |  |
| Have seen a GP for gout in the past 6 months? |  |  | 0.517 |
| Not seen a GP for gout | 57 (18.3) | 28 (16) |  |
| Seen a GP for gout | 254 (81.7) | 147 (84) |  |
| Have seen the specialist for gout in the past 6 months? |  |  | 0.576 |
| Not seen a rheumatologist for gout | 262 (84.2) | 144 (82.3) |  |
| Seen a rheumatologist for gout | 49 (15.8) | 31 (17.7) |  |
| Number of gout attacks in the past 6 months: Median (IQR) | 3 (1-4) | 3 (2-4) | 0.054 |
| Reported taking ULT? |  |  | 0.550 |
| No | 119 (38.3) | 64 (35.6) |  |
| Yes | 192 (61.7) | 116 (64.4) |  |
| Number of tophi: Median (IQR) | 0 (0-1) | 0 (0-2) | 0.227 |
| Most recent gout attack pain (Scale 1-10): Median (IQR) | 7 (6-8) | 7 (5-9) | 0.356 |
| Number of days since last gout attack: Median (IQR) | 36 (9-101.5) | 17 (7-58) | 0.001 |
| Number of days since last gout attack |  |  | 0.006 |
| <100 | 231 (74.8) | 148 (86.5) |  |
| 100-300 | 63 (20.4) | 16 (9.4) |  |
| ≥300 | 15 (4.9) | 7 (4.1) |  |
| Serum urate concentration (mmol/L): Mean (SD) | 0.43 (0.10) | 0.44 (0.11) | 0.329 |
| Target serum urate (SU≤0.36mmol/L) |  |  | 0.623 |
| Not achieved | 233 (74.9) | 159 (76.8) |  |
| Achieved | 78 (25.1) | 48 (23.2) |  |
| Smoking status |  |  | 0.282 |
| Not smoking | 288 (92.9) | 155 (90.1) |  |
| Smoking | 22 (7.1) | 17 (9.9) |  |
| Education |  |  | 0.114 |
| Year 12 and below | 72 (23.2) | 53 (29.6) |  |
| Tertiary education | 239 (76.8) | 126 (70.4) |  |
| Baseline PDC (%): Mean (SD) | 84 (20) | 81 (21) | 0.442 |
| Baseline ULT adherence |  |  | 0.699 |
| Adherent (PDC≥80%) | 45 (66.2) | 25 (62.5) |  |
| Non-adherent (PDC<80%) | 23 (33.8) | 15 (37.5) |  |
| Baseline total ARMS score: Median (IQR) | 15 (13-19) | 16 (13-20) | 0.021 |

BMI = body mass index; GP = general practitioner; IQR = interquartile range; SD = standard deviation; SU = serum urate; ULT = urate-lowering therapy; PDC = Proportion of Days Covered.

Note: All results are presented in n (%) unless otherwise indicated. Patients who responded either ‘I do not know’ or ‘I would rather not respond’ were treated as missing values.
