# Supplementary material for: Psychometric evaluation of the Adherence to Refills and Medications Scale (ARMS) in Australians living with gout
Source: Clin Rheumatol. 2024 Jul 15;43(9):2943–54. doi: 10.1007/s10067-024-07050-y (PMC11330394; doi:10.1007/s10067-024-07050-y)
Supplement: Supplementary file 3 — Supplementary file3 (DOCX 26.9 KB) [file 10067_2024_7050_MOESM3_ESM.docx]

**SUPPLEMENTARY INFORMATION 3**

**Supplementary Table S2** Median, minimum and maximum factor loadings from 25 imputed datasets, assuming data missing at random

|  | **Factor loadings** | | |
| --- | --- | --- | --- |
| **ARMS Items** | **Baseline (n=487)** | **6 months (n=487)** | **12 months (n=487)** |
|  | **Factor 1**  **Median (min-max)** | **Factor 1**  **Median (min-max)** | **Factor 1**  **Median (min-max)** |
| 1: How often do you forget to take your medicine? | 0.423 (0.351-0.517) | 0.341 (0.037-0.789) | 0.344 (-0.287-0.786) |
| 2: How often do you decide not to take your medicine? | 0.457 (0.301-0.571) | 0.428 (-0.202-0.679) | 0.444 (-0.283-0.884) |
| 3: How often do you forget to get prescriptions filled? | 0.387 (0.149-0.514) | 0.541 (0.015-0.749) | 0.360 (-0.054-0.810) |
| 4: How often do you run out of medicine? | 0.481 (0.312-0.653) | 0.461 (-0.146-0.804) | 0.468 (-0.156-0.913) |
| 5: How often do you skip a dose of your medicine before you go to the doctor? | 0.566 (0.499-0.726) | 0.503 (0.034-0.808) | 0.533 (0.274-0.940) |
| 6: How often do you miss taking your medicine when you feel better? | 0.564 (0.461-0.713) | 0.565 (0.230-0.965) | 0.458 (-0.185-0.853) |
| 7: How often do you miss taking your medicine when you feel sick? | 0.495 (0.195-0.632) | 0.470 (-0.254-0.882) | 0.198 (-0.295-0.849) |
| 8: How often do you miss taking your medicine when you are careless? | 0.633 (0.519-0.722) | 0.546 (-0.206-0.909) | 0.530 (-0.164-0.938) |
| 9: How often do you change the dose of your medicines to suit your needs? | 0.583 (0.420-0.689) | 0.530 (-0.114-0.883) | 0.376 (-0.224-0.937) |
| 10: How often do you forget to take your medicine when you are supposed to take it more than once a day? | 0.519 (0.372-0.747) | 0.589 (-0.141-0.911) | 0.468 (-0.165-0.922) |
| 11: How often do you put off refilling your medicines because they cost too much money? | 0.520 (0.407-0.619) | 0.578 (0.129-0.829) | 0.506 (-0.191-0.954) |
| 12: How often do you plan ahead and refill your medicines before they run out? | 0.642 (0.426-0.806) | 0.554 (-0.116-0.916) | 0.609 (-0.049-0.997) |

ARMS = Adherence to Refills and Medications Scale.

**Supplementary Table S3** Median, minimum and maximum factor loadings from 25 imputed datasets, assuming data missing not at random

|  | **Factor loadings** | | |
| --- | --- | --- | --- |
| **ARMS Items** | **Baseline (n=487)** | **6 months (n=487)** | **12 months (n=487)** |
|  | **Factor 1**  **Median (min-max)** | **Factor 1**  **Median (min-max)** | **Factor 1**  **Median (min-max)** |
| 1: How often do you forget to take your medicine? | 0.443 (0.354-0.510) | 0.394 (0.138-0.784) | 0.408 (-0.138-0.771) |
| 2: How often do you decide not to take your medicine? | 0.494 (0.349-0.591) | 0.495 (-0.006-0.714) | 0.544 (-0.073-0.905) |
| 3: How often do you forget to get prescriptions filled? | 0.480 (0.309-0.583) | 0.629 (0.256-0.790) | 0.554 (0.279-0.823) |
| 4: How often do you run out of medicine? | 0.518 (0.393-0.647) | 0.542 (0.076-0.798) | 0.555 (0.020-0.910) |
| 5: How often do you skip a dose of your medicine before you go to the doctor? | 0.602 (0.536-0.751) | 0.585 (0.255-0.818) | 0.639 (0.416-0.953) |
| 6: How often do you miss taking your medicine when you feel better? | 0.604 (0.541-0.732) | 0.631 (0.410-0.953) | 0.574 (0.030-0.853) |
| 7: How often do you miss taking your medicine when you feel sick? | 0.529 (0.292-0.641) | 0.518 (-0.044-0.885) | 0.383 (-0.103-0.862) |
| 8: How often do you miss taking your medicine when you are careless? | 0.659 (0.563-0.744) | 0.603 (-0.001-0.900) | 0.645 (0.034-0.947) |
| 9: How often do you change the dose of your medicines to suit your needs? | 0.600 (0.463-0.676) | 0.574 (0.051-0.877) | 0.561 (-0.064-0.927) |
| 10: How often do you forget to take your medicine when you are supposed to take it more than once a day? | 0.560 (0.461-0.753) | 0.656 (0.100-0.908) | 0.620 (0.071-0.939) |
| 11: How often do you put off refilling your medicines because they cost too much money? | 0.572 (0.484-0.653) | 0.646 (0.334-0.843) | 0.608 (0.023-0.953) |
| 12: How often do you plan ahead and refill your medicines before they run out? | 0.680 (0.533-0.809) | 0.641 (0.146-0.928) | 0.723 (0.107-0.966) |

ARMS = Adherence to Refills and Medications Scale.
